# Effect of Different Vat Polymerization Techniques on Mechanical and Biological Properties of 3D-Printed Denture Base

**DOI:** 10.3390/polym15061463

**Published:** 2023-03-15

**Authors:** Hao-Ern Lee, Muhammad Syafiq Alauddin, Mohd Ifwat Mohd Ghazali, Zulfahmi Said, Syazwani Mohamad Zol

**Affiliations:** 1 Faculty of Dentistry, Universiti Sains Islam Malaysia, Kuala Lumpur 56100, Malaysia; 2 Smart Manufacturing and Advanced Renewable Technology Research Group, Faculty Science and Technology, Universiti Sains Islam Malaysia, Nilai 71800, Malaysia; 3 Department of Conservative Dentistry and Prosthodontics, Universiti Sains Islam Malaysia, Kuala Lumpur 56100, Malaysia; 4 Department of Basic Sciences and Oral Biology, Faculty of Dentistry, Universiti Sains Islam Malaysia, Kuala Lumpur 56100, Malaysia

**Keywords:** three-dimensional printing, vat polymerization, denture base, additive manufacturing, stereolithography, digital light processing, liquid-crystal display

## Abstract

Three-dimensional printing is increasingly applied in dentistry to fabricate denture bases. Several 3D-printing technologies and materials are available to fabricate denture bases, but there is data scarcity on the effect of printability, mechanical, and biological properties of the 3D-printed denture base upon fabricating with different vat polymerization techniques. In this study, the NextDent denture base resin was printed with the stereolithography (SLA), digital light processing (DLP), and light-crystal display (LCD) technique and underwent the same post-processing procedure. The mechanical and biological properties of the denture bases were characterized in terms of flexural strength and modulus, fracture toughness, water sorption and solubility, and fungal adhesion. One-way ANOVA and Tukey’s post hoc were used to statistically analyze the data. The results showed that the greatest flexural strength was exhibited by the SLA (150.8±7.93 MPa), followed by the DLP and LCD. Water sorption and solubility of the DLP are significantly higher than other groups $\left(31.51\pm 0.92\;\frac{\mu g}{mm^{3}}\right)$ and $\left(5.32\pm 0.61\;\frac{\mu g}{mm^{3}}\right),$ respectively. Subsequently, the most fungal adhesion was found in SLA (221.94±65.80 CFU/mL). This study confirmed that the NextDent denture base resin designed for DLP can be printed with different vat polymerization techniques. All of the tested groups met the ISO requirement aside from the water solubility, and the SLA exhibited the greatest mechanical strength.

## 1. Introduction

Edentulism is an irreversible and impairing disease that burdens oral health, affecting many individuals, especially elder adults [1]. A partial or complete denture has been a suitable and effective solution to rehabilitate the patient’s missing dentition. In 1936, polymethyl methacrylate (PMMA) was developed and introduced to prosthetics dentistry. A decade later, in 1948, 98% of dentures worldwide were made from PMMA and copolymers [2]. Heat-polymerized PMMA is an ideal material and has been frequently used for denture fabrication due to its ease of processing, low cost, good aesthetic characteristics, and great biocompatibility [3,4,5]. However, it also possessed multiple issues, such as dimensional inaccuracy, microbial colonization due to porosity, and allergic contact dermatitis caused by uncured monomer [3,6,7].

With the advancement of digital dentistry, computer-aided design and computer-aided manufacturing (CAD/CAM) have been introduced to fabricate dental prostheses including implants and dentures [8,9,10]. Compared to the conventional method of fabrication, the digital method reduces the frequency of patient visit, cost, and human error and increase manufacturing and clinical accuracy [11]. Presently, subtractive manufacturing (SM) and additive manufacturing (AM) is the leading digital fabrication process for denture base. Subtracting manufacturing through computer numeric control (CNC) milling is a process where the dentures are milled off from the industrial prefabrication blank. The subtractive manufactured denture has good mechanical properties and low shrinkage since the polymerization process, such as pre-sintering, was completed before the milling process. Nonetheless, this manufacturing method is also associated with a series of drawbacks, such as high initial cost, wear of milling cutters, and high material wastage of up to 90% [12,13].

In contrast, additive manufacturing, commonly known as 3D printing, is preferable when it comes to fabricating complex geometries such as the denture’s contours. The layer-by-layer working principle allowed 3D printing to manufacture hollowed models and microscopic structures with high precision and accuracy [14]. There is a wide range of additive manufacturing processes capable of printing denture materials. These techniques include fused deposition modeling (FDM), vat polymerization, selective laser sintering (SLS), and poly-jetting. Amongst all the additive manufacturing techniques, vat polymerization is the most favorable and widely used in the fabrication of dentures [15,16]. This is due to its capability to fabricate high-precision dentures rapidly and economically while having an excellent aesthetic.

Vat polymerization, also known as photo-curing 3D printing, is the earliest AM method invented by Charles Hall [17]. This manufacturing method is based on the photo-polymerization technique, where photosensitive resin is activated and cured under light irradiation [18]. With the different principles of pattern illustration and control system, vat polymerization can be divided into numerous techniques, for instance, liquid-crystal display (LCD), continuous liquid interface production (CLIP), stereolithography (SLA), digital light processing (DLP), and other printing techniques [19]. The photo-curing 3D printing has many advantages over other 3D printing technology; for instance, faster fabrication time results in energy saving, great potential in formulating novel hybrid material, and an extensive field of application [20,21]. To date, SLA, DLP, and LCD is the most common and popular manufacturing method to fabricate denture [22,23]. These techniques can produce high-precision dentures rapidly at a lower cost in comparison to SM and traditional methods. In a recent study, the patients reported that the 3D-printed denture base provides good retention and stability, and the overall satisfaction level with the printed denture has drastically improved [24].

The SLA, DLP, and LCD techniques have two configurations, which are the free surface approach (bat configuration) and the constraint surface approach (bath configuration). In the “Bat” configuration machines, they all have a reservoir tank for the photosensitive resin, known as the vat, a build platform that the printed part will adhere onto, and a flexible film at the bottom of the vat so that the printed part can be displaced from the film while still strongly adhered to the build platform. During the printing process, the build platform will move upwards, away from the vat, while the layer forms, and the final part will hang on the build platform upside-down. While in the “bath” configuration, it takes the opposite approach. The light source is located at the top, curing the resin in the vat from above, and the build platform will move inwards into the vat after a layer forms. In this configuration, there is no need for a flexible film since the printed object only cures on the layer before it. However, this configuration has the disadvantage of a chemical reaction with ambient air since the printing layer is in contact with the ambient. Ergo, the “Bat” configuration is increasingly applied in the 3D printing industry compared to the “Bath” configuration [25]. Even in the two configurations, these techniques are comparable in many ways. The main difference is that their mechanism and imaging systems differ from each other. The SLA technique employs an ultraviolet (UV) laser beam as the light source to polymerize the photosensitive resin. With the aid of a mirror and a galvanometer, the UV laser is reflected and directed to the surface of the liquid resin, curing the path of the cross-sectional pattern it traveled [26]. Due to the nature of its working principle, the print volume of an SLA printer can theoretically be as big as possible [27]. However, SLA has a slow printing speed because the curing rate relies on a single moving laser beam. The bigger the printing part, the more time it required to print. While printing multiple parts in a single process is possible, it will take more time to finish.

DLP, on the other hand, employs a high-resolution projector as the light source to cure the resin. Similar to the projector in the theatre, the DLP projector projects an image of the cross-sectional area of the printing part onto the liquid resin, curing the entire cross-sectional layer at a time. This particular projector is called the digital mirror device (DMD). Moreover, the advanced electronic device of DLP allows it to have superior printing resolution. Though, the biggest limitation of this technique is that the build volume was constrained. In order to achieve a high printing resolution, the DLP projector must be placed close to the exposure plane to avoid pixel dilation. Ergo, the DLP technique has the advantage of high printing resolution and printing rate to print smaller parts [19,28].

Lastly, the LCD technique is very similar to the DLP technique; instead of a projector, a liquid-crystal display is employed as the imaging system. While a UV light-emitting diode (LED) array is used as the light source to polymerize the liquid resin, the LCD panel will act as a mask to prevent light from passing through, curing the masked cross-sectional layer all at once [29]. However, during the printing process, a weak light will penetrate through the LCD panel due to a small number of liquid crystal molecules that cannot rearrange. Hence, when it comes to performance and precision, the LCD technique is inferior to the DLP technique. In spite of that, LCD machine is very affordable while having an adequate printing resolution and fast printing rate [30]. Table 1 illustrates the characteristics and differences between the three vat polymerization techniques.

A series of factors can affect the physical, mechanical, and biocompatibility properties of the 3D-printed denture. For instance, the layer height, printing orientation, printing technology, and printing materials [18,31,32,33]. In a recent study [23], the author compared the flexural properties and cytotoxicity of interim materials printed using DLP and LCD technology. It concludes that the photosensitive resin designed for the DLP 3D printers can be printed on an LCD printer with appropriate settings. Moreover, the LCD-printed interim materials have comparable flexural properties in comparison with the DLP-printed interim with a sufficient amount of post-polymerization. In another study [33], the author compared the flexural properties and accuracy of different 3D-printed denture base resins printed on SLA and DLP printers. The results showed that the SLA printed part has the highest accuracy error compared to the DLP, whereas the flexural strength of both was comparable. However, since the author used different proprietary resins for each specific 3D printer, it was uncertain whether the mechanical properties were affected by the material itself or by the printing technology. Furthermore, in a recent study [34], the authors compared the surface hardness of 3D-printed orthodontics aligner material fabricated by LCD and DLP and found that the LCD technique can produce slightly higher hardness. Nonetheless, the current study used a different material than the mentioned study, and varied results were expected. Another similar study compared the surface roughness and hardness of a 3D-printed denture base fabricated with two different DLP machines at different orientations [35]. The results showed that the surface roughness was not affected by the different printers and orientations; however, the different DLP printers did make an impact on the surface hardness. Numerous studies have demonstrated the printing resin, and post-curing setting affect the mechanical properties [4,14,16,18], but no study has thoroughly discussed the different vat polymerization printing technology. To the best of our knowledge, whilst there are studies conducted to characterize the properties of the 3D-printed denture base, there is data scarcity in the current literature in regard to the effect of the 3D-printed denture base printed with different vat polymerization techniques.

Therefore, this study aims to characterize the 3D-printed denture base fabricated using SLA, DLP, and LCD technology in terms of flexural strength and modulus, fracture toughness, water sorption and solubility, and fungal adhesion of Candida Albicans (C. Albicans). The null hypothesis of the present study is that the 3D printing technology will not affect the mechanical and biocompatibility properties of the 3D-printed denture base resin.

## 2. Materials and Methods

### 2.1. Specimens’ Preparation

Commercially available 3D-printed acrylate ester-based resin (NextDent Denture 3D+; 3D Systems, Soesterberg, The Netherlands) was used in this study to determine the mechanical properties and fungal adhesion of the denture base resin fabricated with different vat polymerization techniques.

**Figure 1:**
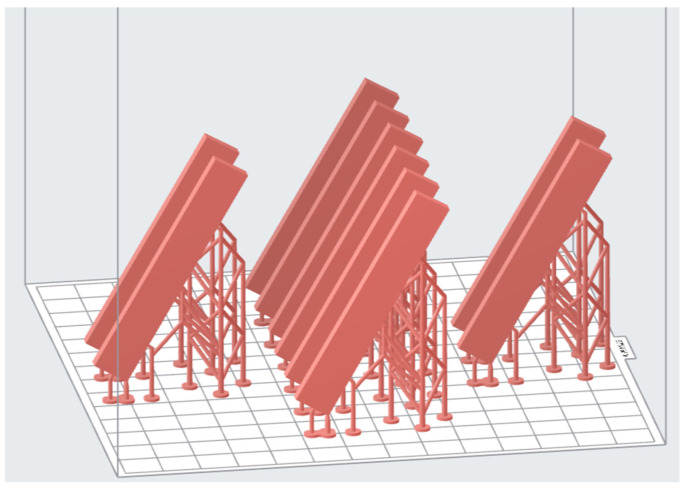
Specimens design exported to slicer.

To characterize the effect of different vat polymerization techniques, the specimens were fabricated using different 3D printers which utilized the SLA, DLP, and LCD techniques. Form 2 (FormLabs, Somerville, MA, USA) was selected to represent the SLA method, NextDent 5100 (NextDent B.V., 3D systems, Soesterberg, The Netherlands) to represent the DLP, and Mono 4K (Anycubic, Shenzhen Anycubic Technology Co., Ltd. Shenzhen, China) to represent the LCD. All the testing specimens were designed using 3D computer-aided design (CAD) software (Fusion 360; Autodesk, San Francisco, CA, USA). Then, the designed specimens were exported into Standard Tessellation Language (STL) file and sliced with the printers’ slicer, respectively. All specimens were printed at a resolution of 50 microns layer height and oriented 45° away from the build plate (Figure 1). For the DLP system, the default setting for the denture base resin was selected in the proprietary slicer. However, in the case of SLA and LCD systems, there are no pre-set settings for the specific denture base resin. Additionally, the printing parameters are not adjustable in the SLA slicer. Hence, for the SLA system, a default setting for its own proprietary dental resin was selected. On the other hand, the LCD slicer had the freedom to adjust the printing parameters such as exposure time, lifting speed, and light-off duration. Ergo, for the LCD system, a custom setting was optimized according to the printability of the denture base resin. This custom setting was trial and error with different exposure times, lifting speeds, and light-off duration and finally optimized with a shorter printing time while at the same time having good printability and high dimensional accuracy. After the printing process, the specimens were removed from the build plate with a metal scrapper and proceeded with the post-rinsing and post-curing process. In the post-rinsing process, the specimens were cleaned in a 99.9% isopropyl alcohol ultrasonic bath for 5 min, according to the manufacturer’s instructions. Then, the support structures of the specimens were removed with a snipper and allowed to air dry for 10 min. Thereafter, the specimens were post-cured in the UV light chamber (LC-3DPrintbox; NextDent) for 30 min. All the specimens were finished using silicon carbide grinding paper consecutively (500, 1000, and 1200 grit) and rinsed with running tap water. The printing parameters, printers, and post-processing procedure are illustrated in Table 2 and Table 3.

### 2.2. Flexural Strength and Modulus

**Figure 2:**
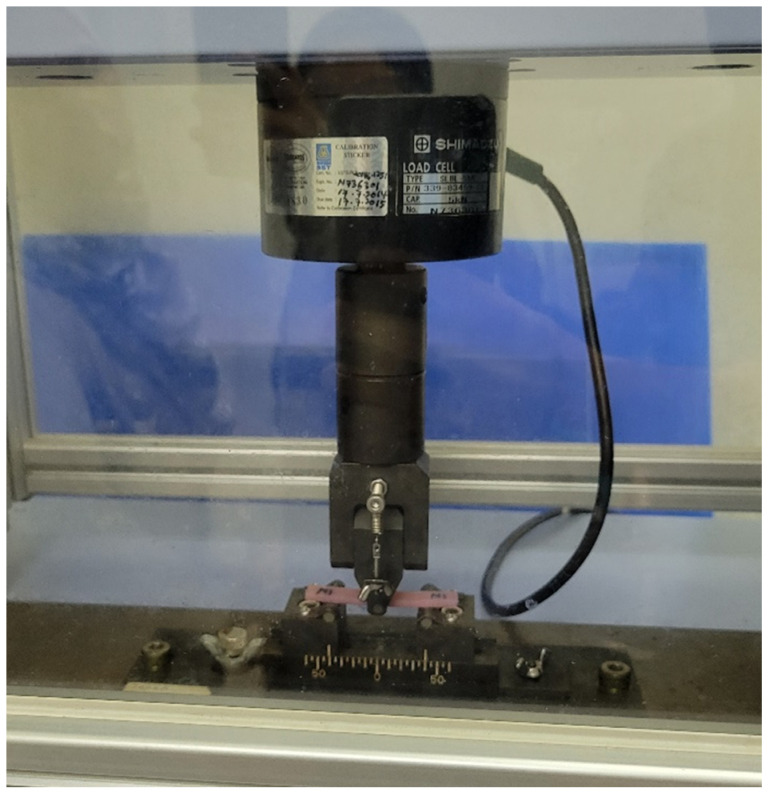
The 3-point bending test on Universal Testing Machine (UTM) (Flexural test).

The 3-point bending test was carried out to determine the flexural strength and modulus of the prepared specimens. The specimens were designed and fabricated in the dimension of (65 mm × 10 mm × 3.3 mm) as recommended in the International Standard Organization (ISO) 20795-1 for denture base polymer [36]. This test was conducted using the Universal Testing Machine (UTM) (SLBL-5kN; Shimadzu, Kyoto, Japan) with the 3-point bending test fixture (Figure 2). All the specimens were stored in distilled water at a temperature of (37 ± 1) °C hours prior to the testing to release residual monomers. Specimens were placed on the fixture with a span width of 50 mm apart, and a constant load of 5 kN was applied at a displacement rate of 5 mm/min until mechanical failure. Further, 10 specimens were prepared for each group for testing (*n* = 10), and a total of 30 specimens were prepared for the 3-point bending test. The maximum load exerted on the specimen was measured, and the ultimate flexural strength (σ) was calculated in MPa using the formula below:

(1) $$\sigma =\frac{3F\ell }{{2bh}^{2}}$$

where F is the maximum load exerted on the specimen, in newtons, ℓ is the span length, b is the width of the specimen, and h is the height of the specimen. Additionally, the material’s resistance to bending force was further characterized with flexural modulus using the formula below:

(2) $$E=\frac{{F\ell }^{3}}{{d\cdot 4bh}^{3}}$$

where *F*/*d* is the gradient of load against the deflection curve at the linear straight-line portion (with the maximum slope) of the curve.

### 2.3. Fracture Toughness

**Figure 3:**
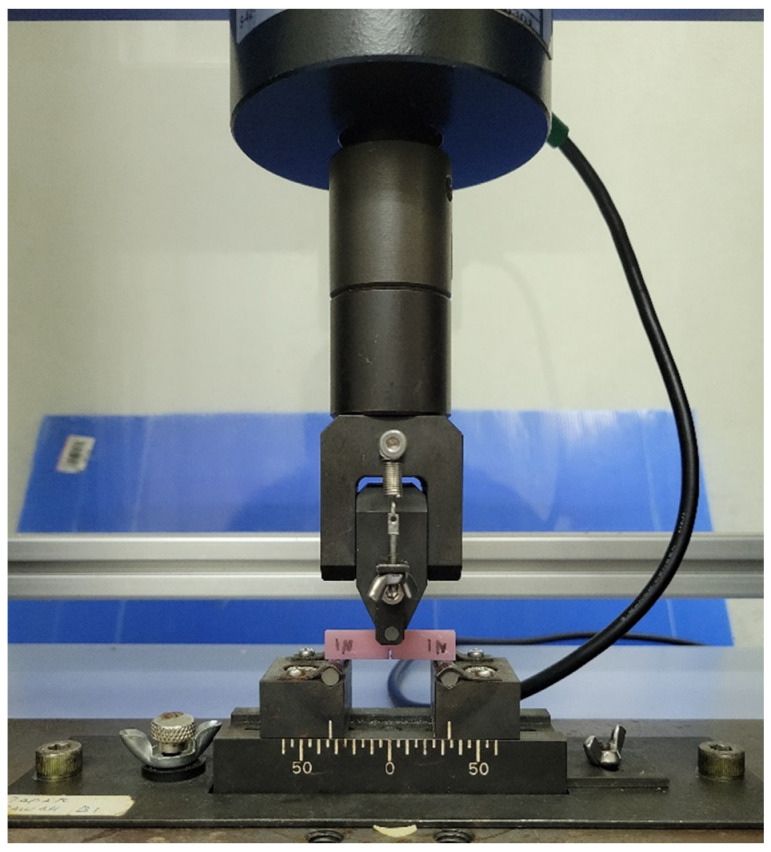
The 3-point bending test on Universal Testing Machine (UTM) (Fracture test).

The fracture toughness was determined using the same method of testing, the three-point bending test, but with a different specimen design and machine-rig setup (Figure 3). The specimens were designed and fabricated in the dimension of (39 mm × 8 mm × 4 mm) with a (3.0 ± 0.2) mm pre-crack in the center, in accordance with the ISO 20795-1 [36]. Specimens were stored in distilled water for (7 d ± 2 h) at (37 ± 1) °C and then conditioned in water at (23 ± 1) °C for (60 ± 15) mins prior to testing. Specimens were placed on a fixture with a span width of 32 mm apart, and a constant load of 5 kN was applied at a displacement rate of 1 mm/min. Then, 10 specimens were prepared for each group for testing (*n* = 10), and a total of 30 specimens were prepared for the fracture toughness test. The maximum force was measured and recorded to calculate the fracture toughness, *K_max_* of the denture base material using the formula below:

(3) $$K_{max}=\frac{fP_{max}l_{t}}{b_{t}{h_{t}}^{3/2}}\times \sqrt{10^{-3}}$$

(4) $$f={3x}^{\frac{1}{2}}\cdot \frac{\left[1.99-x\left(1-x\right)\left(2.15-3.93+2.7x^{2}\right)\right]}{\left[2\left(1+2x\right){\left(1-x\right)}^{\frac{3}{2}}\right]},x=\frac{a}{h_{t}}$$

where *P_max_* is the maximum load exerted on the specimen, in newtons, $b_{t}$ is the width of the specimen, $h_{t}$ is the height of the specimen, $l_{t}$ is the span length, and a is the length of the pre-crack.

### 2.4. Water Sorption and Solubility

**Figure 4:**
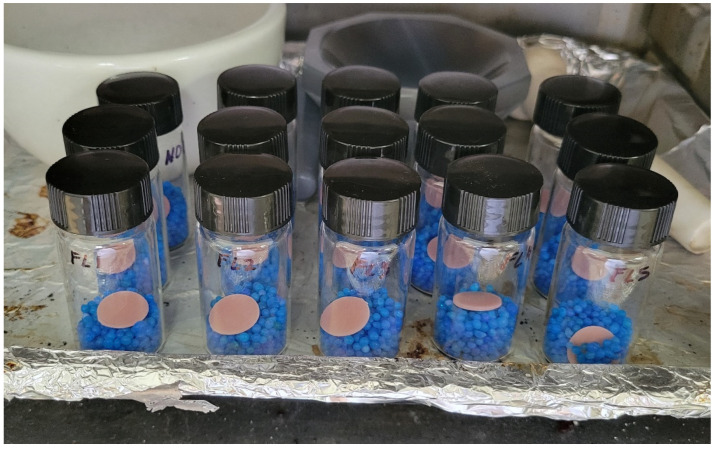
Specimens dehydrating in desiccators in an oven at 37 °C.

Specimens with a diameter of 15 mm and thickness of 1 mm were prepared to determine the water sorption and solubility of the denture base resin fabricated with different vat polymerization techniques [36]. Then, 5 specimens were prepared for each group for testing (*n* = 5), and a total of 15 specimens were prepared for the water immersion test. The specimens were each kept inside a desiccator filled with freshly dried silica gel and placed in an oven for (23 ± 1) h at (37 ± 1) °C (Figure 4) and then transferred to a second desiccator containing freshly dried silica gel at (23 ± 2) °C for another hour. Then, the specimens’ mass was measured with an analytical balance (Mettler ME103E; Greifensee, Switzerland) with an accuracy of 0.1 milligrams. This drying cycle was repeated until the measured mass (*m*_1_) attained a constant mass reading that was not more than 0.2 mg between successive weighing. The mean diameter and thickness of the specimens were measured with a high-accuracy digital caliper at this point in time, and the volume of the specimens (*V*) was calculated. Thereafter, the specimens were transferred into a universal bottle filled with distilled water. The specimens were ensured to be fully immersed in the distilled water at (37 ± 1) °C for (7 d ± 2 h). Then, the specimens were dried, and their weight was measured. This wetting cycle was repeated until a constant mass (*m*_2_) was acquired. Lastly, the specimens were reconditioned in the desiccator mentioned previously in the drying cycle until they reached a final constant mass (*m*_3_). Water sorption $\left(w_{sp}\right)$ and solubility $\left(w_{sl}\right)$ were calculated in $\mu g/{mm}^{3}$ using the formula below.

(5) $$w_{sp}=\frac{m_{2}-m_{3}}{V},w_{sl}=\frac{m_{1}-m_{3}}{V}$$

where $m_{1}$ is the mass of the dried conditioned specimen, $m_{2}$ is the mass of the specimens after being immersed in distilled water, $m_{3}$ is the mass of the reconditioned specimen, and *V* is the volume of the specimen.

### 2.5. Fungus Adhesion

Several microbial quantification methods were available to evaluate microbial adhesion in microbiology; each has its advantages and limitation. Colony-forming units (CFU) counting of the plate is recognized as the gold standard method to quantify the bacteria or fungus cells adhered to a sample [37]. Ergo, the colony formation unit (CFU) assays were conducted to assess the susceptibility of the denture base material to attach fungus. The 3D-printed denture base resin with different fabrication methods (*n* = 4) with a diameter of 10 mm and thickness of 2 mm was fabricated and characterized according to ISO 10993-5 and 10993-12 [38,39]. Candida Albicans (ATCC 18804) (C. Albicans) was incubated in 25 mL Brain Heart Infusion (BHI) broth for 24 h at 37 °C. After that, the fungus from the broth culture was diluted in fresh BHI broth, and the concentration was adjusted to 3 McFarland standards to begin the inoculum process. The specimens were placed in the 12-well plate and inoculated with 100 μL Candida suspension and 1 mL fresh brain–heart infusion medium aerobically for 24 h at 37 °C [40].

**Figure 5:**
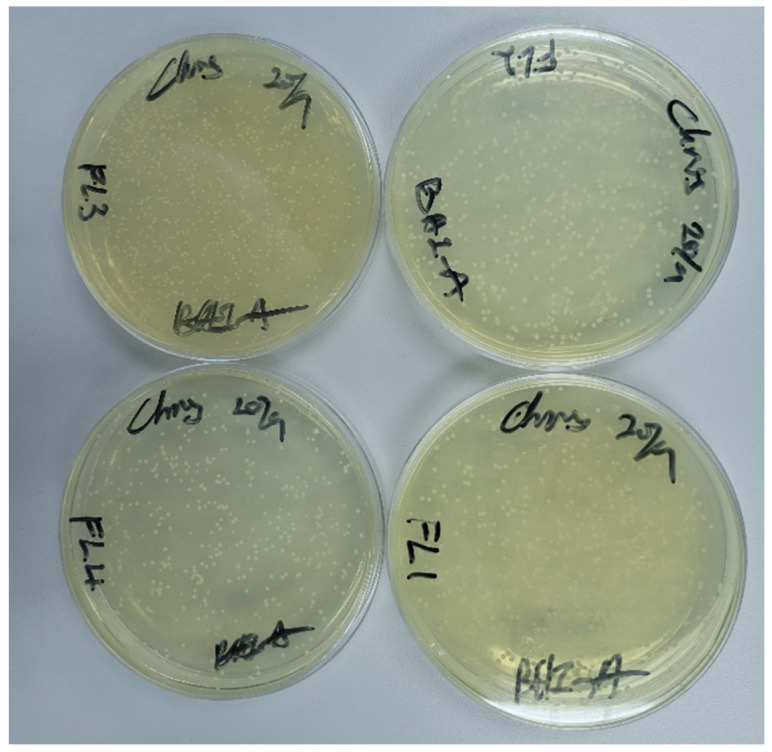
Supernatant of C. Albicans suspension cultured on plate.

After (24 ± 2) hours, the specimens were gently rinsed with phosphate-buffered saline (PBS) to remove the non-adherent cells and transferred to a centrifuge tube filled with 2 mL PBS [41]. To detach the adhered fungus cell from the denture surface, the specimens were vortexed and sonicated for 5 min, respectively. The attained suspension of adherent cells was extracted and diluted to 10^−0.5^ with PBS to prevent cell overgrowth in the Petri dish. Next, 100 μL of the diluted suspension was plated onto BHI agar in the Petri dish and incubated for 24 ± 2 h at 37 °C (Figure 5). The adherence assay was performed in three independent replicates to ensure the repeatability of the testing result [42].

Cell colony numbers were counted on the colony counter, and the number of colony-forming units per mL (CFU/mL) was calculated using the formula below to quantify the fungus adhesion.

(6) $$C=\frac{n_{0}\alpha^{-j}{\alpha_{p}}^{-1}}{V}$$

where *C* is the concentration of the suspension, $n_{0}$ is the counted number of colonies on the plate, α is the dilution factor, $\alpha_{p}$, is the percentage of aliquot plated to culture, and V is the volume of aliquot plated to culture in mL [43].

### 2.6. Statistical Analysis

The collected data were calculated as means and standard deviation. The normality of data distribution was determined using the Shapiro–Wilk test, and the variance homogeneity of the data was confirmed by the Levene test. The data were statistically compared using one-way ANOVA, followed by Tukey’s post hoc statistical analysis according to a significance level set at (α=0.05). All statistical analyses were completed using Statistical Package for the Social Sciences (SPSS v.26; IBM, Armonk, NY, USA).

## 3. Results

### 3.1. Flexural Strength and Modulus

**Figure 6:**
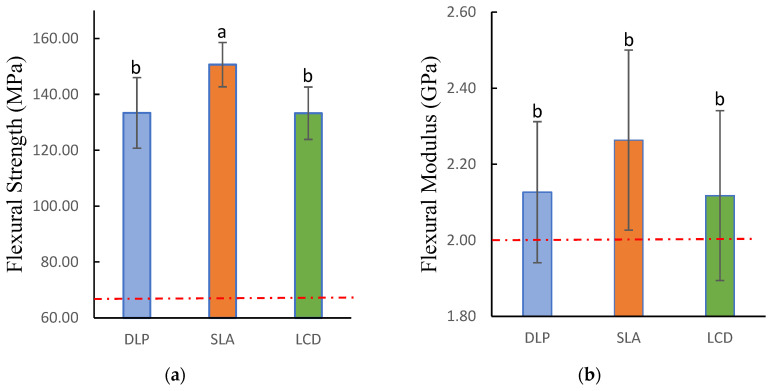
Flexural strength and modulus of 3D-printed denture base resin specimens: (**a**) flexural strength; (**b**) flexural modulus. Similar letters indicate insignificant differences. Red dotted lines indicated the minimum requirements according to ISO standards.

Table 4 presents the testing results for flexural strength and modulus, fracture toughness, water sorption and solubility, and fungal adhesion. One-way ANOVA proved that the flexural strength was significantly different amongst all groups (p<0.001). Additionally, all the groups had exceeded the minimum requirement of 65 MPa according to the ISO standard [36]. The highest flexural strength was exhibited by the SLA group with a mean of (150.68±7.93) MPa, followed by DLP (133.39±12.66) MPa, and LCD (133.28±9.39) MPa. The SLA group had significant differences with both DLP and LCD groups (p=0.002), while there was no significant difference was observed between the LCD and DLP groups. On the other hand, the flexural modulus showed no significant difference between all groups (p=0.256). Nonetheless, all groups had surpassed the minimum requirement of 2 GPa indicated in the ISO standard. The acquired data were illustrated in Figure 6.

### 3.2. Fracture Toughness

**Figure 7:**
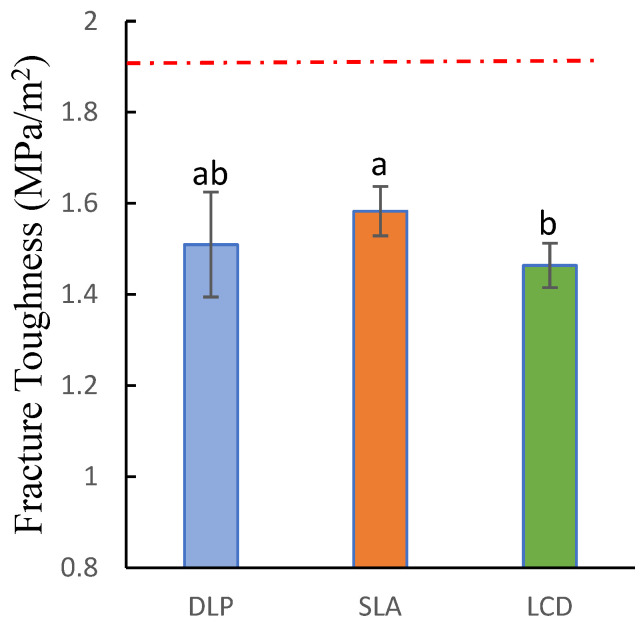
Fracture toughness of 3D-printed denture base resin specimens. Similar letters indicate insignificant differences. Red dotted line indicated the minimum requirement according to ISO standards.

As shown in Figure 7, evaluation through the one-way ANOVA showed that fracture toughness $\left(K_{max}\right)$ had statistically significant differences among all groups (p=0.013). Similar to the flexural strength, the SLA group demonstrated the highest fracture toughness among all groups $\left(1.51\pm 0.115\right)\;MPa/m^{2}$, while the LCD group showed the lowest $\left(1.464\pm 0.049\right)\;MPa/m^{2}$. There is no significant difference observed between LCD and DLP group (p=0.446) and between DLP and SLA group (p=0.14). Notwithstanding, SLA has significantly differed from the LCD group (p=0.01). Additionally, none of the tested groups had met the minimum requirement according to the ISO recommendations.

### 3.3. Water Sorption and Solubility

**Figure 8:**
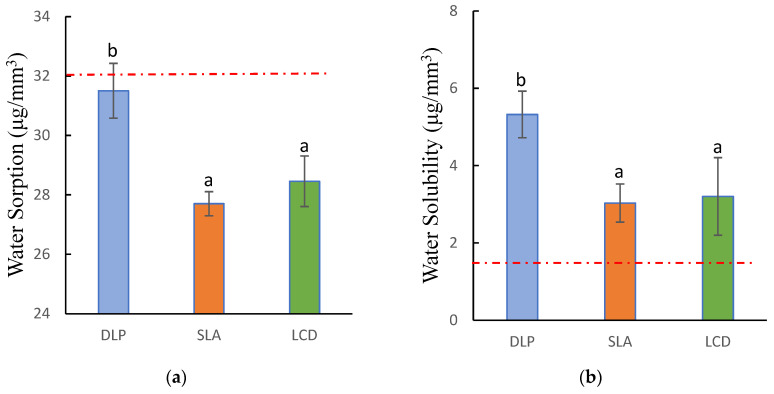
Water sorption and solubility of 3D-printed denture base resin: (**a**) water sorption; (**b**) water solubility. Similar letters indicate insignificant differences. Red dotted lines indicated the maximum requirements according to ISO standards.

The water sorption $\left(w_{sp}\right)$ of all 3D-printed specimens met the requirements of the ISO standard of 32$\mu g/{mm}^{3}$; however, none of the groups met the requirement for water solubility $\left(w_{sl}\right)$. Both water sorption and solubility were statistically significant among all groups (p<0.001). The DLP group demonstrated the highest water sorption and solubility $\left(31.51\pm 0.92\right)\;\mu g/{mm}^{3}$ and $\left(5.32\pm 0.61\right)\;\frac{\mu g}{{mm}^{3}},$ respectively, and were significantly higher than the LCD and SLA groups. On the other hand, there were no significant differences between LCD and SLA (p=0.929). The SLA showed the least water sorption and solubility $\left(27.7\pm 0.4\right)\;\mu g/{mm}^{3}$ and $\left(3.03\pm 0.49\right)\;\frac{\mu g}{{mm}^{3}},$ respectively, while the LCD was slightly higher $\left(28.46\pm 0.38\right)\;\mu g/{mm}^{3}$ and $\left(3.2\pm 1\right)\;\mu g/{mm}^{3}$ (Figure 8).

### 3.4. Fungal Adhesion

**Figure 9:**
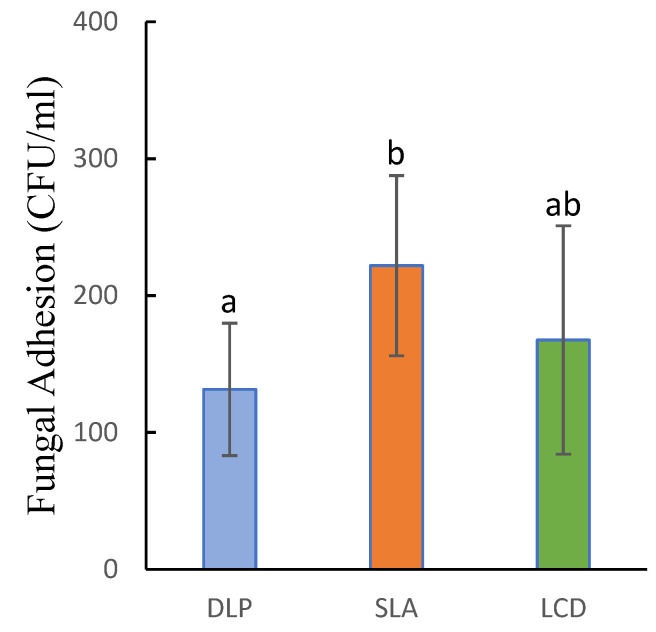
Adherence of Candida Albicans on 3D-printed denture base resin specimens. Similar letters indicate insignificant differences.

The adhesion of C. Albicans on 3D-printed dentures showed a statistically significant difference and was confirmed by the one-way ANOVA (p=0.029). In this adherence test, the DLP group exhibited the strongest antifungal activity with the least C. Albicans attached to the surface of the specimens $\left(131.61\pm 48.38\right)\;CFU/mL$. Subsequently, LCD showed slightly higher adhesion $\left(167.89\pm 83.1\right)\;CFU/mL$, while the FL had the highest fungal adhesion $\left(221.94\pm 65.8\right)\;CFU/mL$. There was no significant difference observed between DLP and LCD (p=0.497) and between SLA and LCD (p=0.224). However, significant differences were reported between DLP and SLA (p=0.023). Figure 9 shows the detailed results of the C. Albicans adherence on the 3D-printed specimens.

## 4. Discussion

This present study elucidated the printability and characterization of the 3D-printed denture base resin material fabricated by 3D printing with different vat polymerization techniques. Stereolithography (SLA), digital light processing (DLP), and liquid-crystal display (LCD) is the most practice technique in vat polymerization to fabricate dentures. Although these techniques work under the same principle of photo-polymerization, their imaging system differs from each other and might result in different printing outcome. Moreover, the commercially available 3D printing resins were only designed for a particular printing technique. For instance, the NextDent Denture 3D+ is a 3D printing denture resin material that is a part of the NextDent close system that only allows printing with its own proprietary printer. This study elucidated that the flexural strength, fracture toughness, water sorption and solubility, and fungal adhesion deviated when the denture base material was fabricated with different vat polymerization techniques. Henceforth, the null hypothesis of this study was rejected.

This study confirmed that the denture base resin that was designed for the DLP 3D printers can be printed in different vat polymerization technique printers, as long as the light source of the machine can emit the appropriate wavelength of light to activate the resin material. Moreover, the liquid resin would also require an appropriate combination of light exposure time and intensity to be polymerized.

During mastication, the denture base will experience a series of compressive and shear stress, which could ultimately lead to the failure of the denture. Ergo, it is critical to ensure that the denture has sufficient mechanical strength to resist fracture and deformation under the mastication load [44]. The flexural strength was described as the denture base material’s performance under the mastication load, while the flexural modulus was associated with elastic deformation [45]. This study concluded that the SLA technique had achieved the highest overall mechanical strength. In regard to the flexural strength and fracture toughness, the SLA technique achieved exceptional results, which is the greatest amongst the tested vat polymerization techniques. This result was in line with the previous study, where the SLA technique can produce a higher strength denture than the DLP technique, while the LCD technique has a comparable strength to the DLP technique [23,33,46]. Nonetheless, the DLP technique has adequate results in mechanical strength, meeting the ISO standard for flexural strength and modulus. The LCD technique, on the other hand, has similar flexural strength and modulus to the DLP technique. It is worth noting that when it comes to fracture toughness, all 3D-printed specimens did not satisfy the minimum criteria of $1.9\;MPa/m^{2}$ in accordance with the ISO standard. However, this criterion is only for that denture resin that claims to have improved impact resistance, and the selected denture base resin did not claim as such. A similar result was presented in a previous study where the fracture toughness of the 3D-printed resin was inferior when compared to the conventional denture base resin and did not meet the ISO recommendation [47]. There are a series of factors that could affect the mechanical strength of the 3D-printed resin material, such as degree of conversion (DC), formation of voids within the material, and anisotropy of printed objects [22,48,49]. The fast process of layer-by-layer mechanism leads to insufficient intensity of curing at each layer and eventually reduces the overall chain crosslinking. Hence, there are monomers that remain unreacted within the 3D-printed part [16]. To address this issue, post-curing was introduced to further convert the unreacted monomers into the polymer and hence improve the degree of conversion. In this case, since all the specimens had the exact same print settings and post-processing procedure, it may be explained by the exposure under the UV light during the printing process had varied the DC. During the printing process, the monomers and photo-initiator in the liquid resin will be activated by the UV light, converting the monomer into the polymer and producing bonded chains at the macromolecular level. DC is the percentage of monomers had been activated and converted into polymers and are affected by light exposure time, light intensity, and temperature [48,50].

In relation to the result of mechanical testing, it is worthwhile to note that the printing time could affect the final result. The SLA technique required the most printing time to fabricate the specimens. The time required for the DLP, LCD, and SLA groups to fabricate five flexural test specimens was 75 min, 90 min, and 250 min, respectively. This showed that the SLA has the slowest printing rate due to its imaging system mechanism of curing photosensitive resin as the laser travels, the light travels on the cross-sectional layer of the printing part was longer in comparison to the LCD and DLP technique, where the entire layer was cured at once. Although SLA had a lower light intensity, the additional exposure time resulted in a higher DC [51]. There is a direct relationship between the light intensity and exposure time toward the degree of conversion. The higher light intensity and longer exposure time will result in higher DC [22,52]. However, overpowering the light intensity during the printing process might cause an overcuring of the resin in the vat, which could lead to dimensional inaccurate and deterioration in mechanical properties. Hence, the optimized combination of light intensity and exposure time can ensure the printed part has adequate mechanical properties while maintaining short fabrication time and high dimensional accuracy. As mentioned above, the LCD and DLP had similar mechanical strength. This may be attributed to the compensation for exposure time during the printing process. The LCD had the lowest light intensity among all groups; to address this issue, it had to increase the exposure time per layer. As a result, the printing rate of the LCD is slower when compared to the DLP. Even though the flexural strength and modulus of LCD and DLP had no significant difference, the higher light intensity of the DLP has brought the advantage of shorter overall printing time. Regarding the lower mechanical properties of DLP when compared to the SLA, the proprietary DLP system optimized the printing parameters accordingly to achieve a shorter fabrication time. In other words, the exposure time was tuned down in order to shorten the fabrication time. Ergo, a decrease in mechanical strength was expected. Nonetheless, the flexural strength and modulus of the DLP control group are still within the ISO recommendation. Additionally, there is another possibility that could affect the DC during the printing process, which is the method of light projection. In the SLA technique, the resin polymerized as the UV laser traveled. In comparison, the DLP and LCD are through a projection of a pixelated pattern. The differences in the projection method might also influence the monomer conversion throughout each layer and, as a result, affecting the mechanical properties of the printed parts.

Since dentures reside in the oral cavity and are always bathed in saliva and other oral fluid, water sorption and solubility are one of the utmost critical factors that could negatively affect the durability and comfortability of the denture [2,53]. Water absorption is the nature of polymeric materials due to their inherent polarity [54]. Smaller-scale water molecules can penetrate and diffuse into the polymer network macrostructure; as a result, degrading and breaking down the resin matrix chemical bond, consequently affecting the polymer solubility [55]. Additionally, the water absorbed can further induce issues such as material softening, reduction in strength and flexibility, and deformation due to dimensional deviation [56]. Henceforth, it is essential for the denture base material to have water sorption and solubility as low as possible. This study revealed that the specimens prepared by the SLA had the lowest sorption rate, mainly due to a higher monomer conversion rate. The higher degree of polymerization resulted in lesser unreacted monomers and therefore led to lower water sorption [57]. The results for the sorption test also corresponded to the mechanical strength, as the SLA technique had the highest mechanical strength while having the lowest water sorption. During immersion in water, unreacted monomers, initiators, and water-soluble elements will leach out from the polymeric material; this is also known as solubility. In a previous study, the authors reported a correlation between water sorption and solubility. Materials with high solubility will tend to absorb more water [58]. This finding was in line with it; the specimens prepared by the DLP had illustrated high water solubility and sorption, respectively. Regardless of the differences, all the specimens met the requirement stated in ISO 20795 for water sorption, less than $32\;\mu g/{mm}^{3}$. However, for the solubility, none of the groups sufficed the requirement in accordance with the standard, $1.6\;\mu g/{mm}^{3}$. This finding is in line with the previous study, where the 3D-printed specimens did not meet ISO requirements before aging. However, the water solubility decreased and met the ISO recommendation after aging in tempered water [59]. This might be the result of residue monomers having dissolved out of the material during the aging process, and hence the mass change within the material was decreased. In the current study, the specimens did not experience any aging process and began with the water immersion test at the initial state. Therefore, the residue monomers within the material would cause an increase in the water solubility.

Fungal adhesion is an important characteristic when it comes to dental prostheses, especially dentures, as it could contribute to the accumulation of dental plaque. Candida Albicans is a major causative agent that induces denture stomatitis, and the removal of C. Albicans has proved to be effective in treating and inhibiting denture stomatitis [60]. This study showed that the denture base material prepared by the SLA had the most C. Albicans adhesion, and this can be attributed to the SLA polymerization mechanism. Instead of polymerizing the entire layer at once, such as DLP and LCD, SLA polymerizes the liquid resin with a moving laser, curing the cross-sectional area line by line. This might cause an irregular surface on the denture, allowing the C. Albicans to adhere to the surface. In contrast, the DLP and LCD with the imaging system of curing the entire layer at once had better performance in inhibiting the adherence of the C. Albicans. An existing study compares the surface roughness between two DLP techniques and the heat-polymerized material [35]. The study showed that the surface roughness did not have significant changes across the two DLP techniques when printing with different orientations and post-curing with different time periods. This finding is, to a certain degree, in line with the current study. The LCD and DLP used a similar mechanism of curing the cross-sectional layer at once; hence, the surface roughness was comparable. In contrast, the SLA mechanism of curing the cross-sectional layer line-by-line would result in higher surface roughness. This finding is in accordance with a previous study [61], where the author compared the surface roughness of the DLP and SLA-printed dental splint and found that the DLP-printed part had a lower surface roughness. Nonetheless, according to a previous study, the layered structure and micro-pores caused by additive manufacturing can be removed after sufficient mechanical polishing [2]. Hence, the irregular surface could be resolved with a more thorough post-mechanical finishing and polishing. The findings of the current study were matched with a previous study, where the author compared the C. Albicans adhesion on conventional, milled, and 3D-printed dentures [62]. The results showed that in the 3D printing group, the DLP specimens have relatively lesser C. Albicans adherence compared to the SLA specimens. However, due to the lack of sample size, the results showed that the differences were insignificant. Notably, the results of this study were in contradiction with another study where the authors stated that there was no effect on the C. Albicans adhesion when fabricating with DLP and SLA techniques [14]. Fungal adhesion to denture base material can vary greatly due to many factors, such as the concentration of fungus in the testing environment, different experiment setups, resin material, and post-processing [14,63]. Hence, there might be a contradiction when comparing the results with the previous study.

Digital technology has been advancing exponentially in recent years; progressively, 3D printing has been introduced to the dental field. At present, vat polymerization is the most frequently employed 3D printing technology in dentistry. Knowledge of the potentiality in terms of feasibility and reliability of this 3D printing technology can increase and improve the use of this technology in various industries, especially in dentistry. This study illustrated a wide range of materials can be used within the different vat polymerization technique by demonstrating the use of Denture 3D+, a photopolymer that was designed for the DLP system. Moreover, the denture material fabricated by the three common vat polymerization techniques was investigated in this study to elucidate the difference between their performance. The limitations of the present study were that only one type of 3D printing denture base resin was used; these results might differ when involving different brands and types of 3D printing resin. Ergo, different 3D printing resins could be used, and characterization such as surface roughness, surface hardness, and degree of conversion can be conducted in the future.

## 5. Conclusions

Within the limitation of the present study, it can be concluded that with the appropriate wavelength of light, the 3D printing resin designed for the DLP printer can be printed in different vat polymerization techniques for 3D printers. In addition, the SLA technique demonstrated exceptional performance in mechanical properties in terms of having the highest flexural strength and fracture toughness and the lowest water sorption and solubility due to a higher degree of conversion. However, in exchange for the greater mechanical properties is the slower printing rate and higher fungal adhesion. Furthermore, although the LCD did not demonstrate exceptional results, it is still acceptable and in accordance with the ISO standard, similar to DLP and SLA.

## Figures and Tables

**Table 1:** Schematic diagrams and characteristics of different vat polymerization techniques.

SLA	DLP	LCD
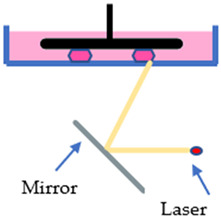	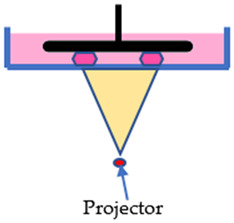	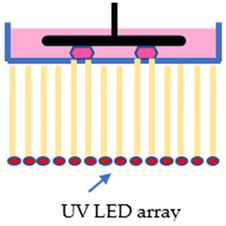
Cross-sectional layer is cured line by line	Entire cross-sectional layer is cured at once	Entire cross-sectional layer is cured at once
Decent precision	High precision	Mediocre precision
Scalable build volume	Limited build volume	Mediocre build volume
Low print rate	Fast print rate	Mediocre print rate

**Table 2:** Three-dimensional printers to represent the different vat polymerization techniques.

Printing Technique	3D Printer	Abbreviation	Manufacturer
Digital Light Processing	NextDent 5100	DLP	NextDent B.V., Soesterberg, The Netherlands
Liquid-Crystal Display	Mono 4K	LCD	Shenzhen Anycubic Technology Co., Ltd. Shenzhen, China
Stereolithography	Form 2	SLA	Formlabs Inc., Somerville, MA, USA

**Table 3:** Printing and post-processing parameters.

Group	Layer Thickness	Wavelength || Light Intensity	Post-Washing Solution	Post-Washing Duration	Post-Curing Station	Post-Curing Duration
DLP	50 microns	405 nm || 1.4 mW/cm^2^	Ultrasonicate in 99.9% IPA	5 min	LC-D Print Box (3D systems)	30 min
LCD	50 microns	405 nm || 1.09 mW/cm^2^	Ultrasonicate in 99.9% IPA	5 min	LC-D Print Box (3D systems)	30 min
SLA	50 microns	405 nm || 1.176 mW/cm^2^	Ultrasonicate in 99.9% IPA	5 min	LC-D Print Box (3D systems)	30 min

**Table 4:** Flexural strength (*σ*), flexural modulus (E), fracture toughness (*K_max_*), water sorption (*w_sp_*), water solubility (*w_sl_*), and C. Albicans adhesion (*C*) results of 3D-printed denture base printed with different vat polymerization techniques.

Characterization	NextDent 5100 (DLP)	Formlabs Form2 (SLA)	Anycubic Mono 4K (LCD)	ANOVA
	Mean ± (SD)	Mean ± (SD)	Mean ± (SD)	*F*- and *p*-Value
Flexural Strength (MPa)	133.39 (12.66) ^b^	150.68 (7.93) ^a^	133.28 (9.39) ^b^	*F* = 9.664
				*p* = 0.001 *
Flexural Modulus (GPa)	2.127 (0.186) ^b^	2.264 (0.237) ^b^	2.118 (0.224) ^b^	*F* = 1.433
				*p* = 0.256
Fracture Toughness (MPa/m^2^)	1.51(0.115) ^ab^	1.583(0.054) ^a^	1.464(0.049) ^b^	*F* = 5.243
				*p* = 0.013 *
Water Sorption (μg/mm^3^)	31.51 (0.92) ^b^	27.7 (0.40) ^a^	28.46 (0.38) ^a^	*F* = 35.021
				*p* = 0.000 *
Water Solubility (μg/mm^3^)	5.32 (0.61) ^b^	3.03 (0.49) ^a^	3.2 (1.00) ^a^	*F* = 15.091
				*p* = 0.001 *
C. Albicans Adhesion (CFU/mL)	131.61 (48.38) ^a^	221.94 (65.80) ^b^	167.89 (83.10) ^ab^	*F* = 4.109
				*p* = 0.029 *

* Statistically significant at 0.05 level. Means with the same superscripts are statistically indifferent.

## Data Availability

Not applicable.
